# Spontaneous renal tumour regression following an aortic dissection

**DOI:** 10.1308/rcsann.2022.0134

**Published:** 2023-01-09

**Authors:** J Schamschula, S Young, S Pridgeon

**Affiliations:** ^1^ James Cook University, Cairns, Australia; ^2^ Cairns Hospital, Australia; ^3^ Northern Urology, Cairns, Australia; ^4^ Australian Clinical Trials and Research, Cairns, Australia

**Keywords:** Carcinomas, Renal cell, Neoplasm regression, Spontaneous, Infarction, Aneurysm

## Abstract

Spontaneous tumour regression is a rare but well-documented phenomenon, especially for renal cell carcinomas. We describe the case of a 60-year-old male who presented with chest pain and shortness of breath. He was diagnosed with a large type A aortic dissection and an incidental right renal mass, highly suspicious of a renal cell carcinoma. Following repair of the dissection, subsequent imaging showed that the renal mass had largely resolved. Spontaneous tumour regression is commonly thought to occur through immunological mechanisms. A vascular cause of tumour regression through infarction is postulated in this case. Although angioembolisation is a well-recognised management option in the context of palliative treatment of symptomatic renal tumours, this case suggests an extended role for angioembolisation in the treatment of small renal masses.

## Background

Spontaneous tumour regression is the complete or partial resolution of a tumour without treatment, or in the presence of inadequate therapy.^1^ Renal cell carcinomas (RCC) have a higher rate of spontaneous regression compared with other cancers and regression is reported in approximately 1% of cases.^1^ Several studies report regression of RCC metastases following primary tumour treatment; this phenomenon is often postulated to have an immunological basis.^1^ This case study describes the regression of a primary tumour through an ischaemic mechanism.

## Case history

**Figure 1:**
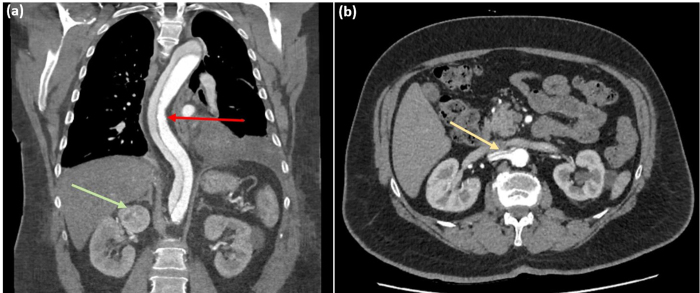
(a) Arterial phase computed tomography scan of chest and upper abdomen demonstrating a type A aortic dissection (red arrow). The dissection extends from below the aortic root to below the level of the renal arteries. A 47×37×46mm mass lesion, highly suspicious of a renal cell carcinoma is incidentally found on the upper pole of the right kidney (green arrow). (b) The true and false lumens of the aortic dissection extend into the right renal artery (yellow arrow).

A 60-year-old male presented to a rural hospital with chest pain and shortness of breath on a background of obesity, hypertension and left ventricular hypertrophy. Examination revealed a blood pressure of 183/70mmHg and no chest findings. Workup was negative for acute myocardial infarction. A computed tomography (CT) scan of the T chest abdomen pelvis (Figure 1a) showed a type A aortic dissection from the aortic root extending proximally into the pericardium and distally into the right renal artery (Figure 1a). A 47×37×46mm tumour was found on the upper pole of the right kidney (Figure 1a). There was no evidence of metastatic disease. The patient underwent emergent surgical repair of the aortic dissection.

A multiphase CT scan of the kidneys at one month, showed a round, heterogeneous, enhancing mass in the right kidney. There was a measurable reduction in diameter by 5mm. In keeping with local protocol, a biopsy of the lesion was not performed. The multidisciplinary team meeting concluded the most likely diagnosis was an RCC. There was no detectable macroscopic fat within the lesion. The differential diagnoses considered included a renal abscess, which was deemed unlikely given there was no ring-enhancing lesion and a lack of perinephric stranding. A renal angiomyolipoma was also considered but thought to be unlikely due to the lack of fat demonstrated on CT. Given the high likelihood that the lesion was malignant, the patient was offered a partial nephrectomy; however, he opted for surveillance while recovering from the aortic dissection surgery.

**Figure 2:**
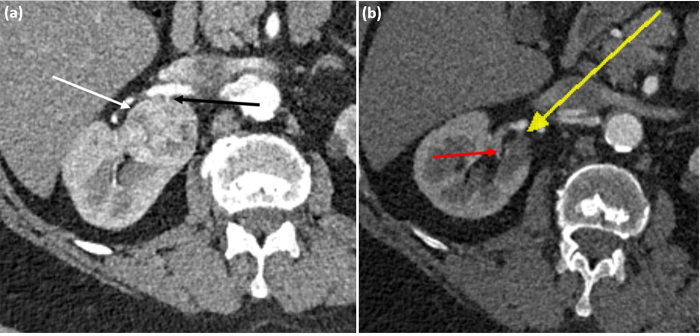
(a) Computed tomography (CT) scan showing renal mass (white arrow) with a small enhancing feeding vessel (black arrow) arising from renal artery. (b) CT scan of right kidney at 11 months after the initial presentation demonstrates the renal lesion had largely regressed (red arrow) and the feeding vessel no longer enhanced on arterial phase imaging (yellow arrow).

A CT scan undertaken 11 months later revealed the right renal mass had largely regressed (Figure 2b), measuring 14mm in diameter. The dissection flap in the right renal artery persisted. On retrospective review of the initial scans, a previously seen feeding vessel to the lesion was no longer enhanced on arterial phase imaging (Figure 2a,b). It was concluded that the renal artery dissection compromised the tumour feeding vessel with thrombosis and infarction, causing spontaneous tumour regression. Two years following the initial presentation, the patient is well and is under ongoing surveillance with no evidence of tumour growth.

## Discussion

This case report documents the regression of a primary renal mass due to infarction. It appears the tumour was preferentially infarcted whereas the remainder of the right kidney enhances normally. We propose several theories to explain this. First, the radius of the tumour feeder vessel is significantly less than that of the renal artery. As per Poiseuille’s relationship, this decrease in radius diminishes flow to the fourth power, thus significantly decreasing blood flow to the tumour compared with the rest of the kidney. Second, tumour neovascularisation is highly aberrant in structure and function. This may make the tumour feeder vessel disproportionately prone to embolic processes compared with healthy arteries, explaining preferential embolisation of the tumour. Lastly, the dissection or infarction may have stimulated an inflammatory response, acting as a sensitising event for an anti-tumour immunological response. RCCs are immunogenic tumours, which may explain their higher rates of spontaneous regression.^1^ It is conceivable that a combination of these explanations accounts for the spontaneous regression of the renal tumour.

Spontaneous renal tumour regression due to vascular pathology has been described previously following haemorrhage into a tumour and in association with renal vein emboli.^1^ Sammut *et al* document a case of complete renal tumour regression following ligation of a tumour feeder vessel during the surgical repair of an abdominal aortic aneurysm.^2^

Small renal masses are increasingly detected on routine abdominal imaging.^3^ This has generated increased interest in minimally invasive procedures such as radiofrequency ablation, percutaneous cryotherapy and stereotactic radiotherapy to treat small kidney tumours.^3^ The European Association of Urology guidelines conclude that minimally invasive ablative techniques are safe and associated with good long-term survival; however, the current data are inadequate to support their benefit compared with surgery.

Angioembolisation involves selective arterial occlusion by embolic agents such as coils, plugs, sclerosants or particles.^4^ Angioembolisation is utilised to manage local symptoms such as haematuria in patients with non-curable disease or in patients who are unsuitable for surgical management.^3^ There are no randomised control trials comparing angioembolisation with nephrectomy for the management of renal masses.^3^ There are, however, several retrospective studies showing promising results.^3^ Techniques that preserve renal function are particularly important in the context of globally increasing mortality from chronic kidney disease.^5^

## Conclusions

This case illustrates a novel mechanism of spontaneous renal tumour regression infarction following renal artery dissection. In an era in which nephron-sparing techniques are preferred for the management of renal tumours, targeted angioembolisation following detailed vascular imaging may offer a potential therapeutic opportunity to achieve cytoreductive tumour ablation.

## Author contributions

Conceptualisation, original draft writing, manuscript editing and review: J.S. Supervising consultant, manuscript editing and review: S.P. Radiological guidance, image selection, manuscript editing and review: S.Y.
